# A high-density exome capture genotype-by-sequencing panel for forestry breeding in *Pinus radiata*

**DOI:** 10.1371/journal.pone.0222640

**Published:** 2019-09-30

**Authors:** Emily Telfer, Natalie Graham, Lucy Macdonald, Yongjun Li, Jaroslav Klápště, Marcio Resende, Leandro Gomide Neves, Heidi Dungey, Phillip Wilcox

**Affiliations:** 1 New Zealand Forest Research Institute LTD. trading as Scion, Rotorua, New Zealand; 2 Horticultural Sciences, University of Florida, Gainesville, FL, United States of America; 3 RAPiD Genomics LLC, Gainesville, FL, United States of America; 4 Department of Mathematics and Statistics, University of Otago, Dunedin, New Zealand; Instituto Nacional de Investigacion y Tecnologia Agraria y Alimentaria, SPAIN

## Abstract

Development of genome-wide resources for application in genomic selection or genome-wide association studies, in the absence of full reference genomes, present a challenge to the forestry industry, where longer breeding cycles could benefit from the accelerated selection possible through marker-based breeding value predictions. In particular, large conifer megagenomes require a strategy to reduce complexity, whilst ensuring genome-wide coverage is achieved. Using a transcriptome-based reference template, we have successfully developed a high density exome capture genotype-by-sequencing panel for radiata pine (*Pinus radiata* D.Don), capable of capturing in excess of 80,000 single nucleotide polymorphism (SNP) markers with a minor allele frequency above 0.03 in the population tested. This represents approximately 29,000 gene models from a core set of 48,914 probes. A set of 704 SNP markers capable of pedigree reconstruction and differentiating individual genotypes were tested within two full-sib mapping populations. While as few as 70 markers could reconstruct parentage in almost all cases, the impact of missing genotypes was noticeable in several offspring. Therefore, 60 sets of 110 randomly selected SNP markers were compared for both parentage reconstruction and clone differentiation. The performance in parentage reconstruction showed little variation over 60 iterations. However, there was notable variation in discriminatory power between closely related individuals, indicating a higher density SNP marker panel may be required to elucidate hidden relationships in complex pedigrees.

## Introduction

Radiata pine (*Pinus radiata* D.Don) is the most widely planted exotic conifer species in the world [1]. In New Zealand nearly 90% of the 1.8 million hectares of stocked plantation forest area is radiata pine, and this represents the third largest export earner in New Zealand at $4.8 billion per annum [2]. While the current radiata pine breeding programme is in its third generation, having been initiated in the 1950s [3], there is increasing pressure on the breeding programme to accelerate the delivery of genetic gain for this economically important species. The development of high density genome-wide single nucleotide polymorphism (SNP) marker resources is key to a number of downstream applications of interest to the forestry industry [4]. Early prediction of genomic breeding values [5], particularly for traits with late expression in long-lived forest tree species [6, 7], monitoring of relatedness within breeding programmes [8, 9] and pedigree reconstruction in open-pollinated trials [10], are but a few of the possible applications. We have chosen to develop a genome-wide marker panel suitable for several downstream applications requiring anywhere from several hundred, to up to many thousands of markers, and to test the efficacy initially in pedigree reconstruction.

Pedigree reconstruction is a popular tool utilising genomic resources into operational breeding programmes and allows the true pedigree of individual trees from within open-pollinated or mixed seedlots to be predicted cost-effectively. This can minimise the need for expensive and time-consuming control-pollinated breeding programmes in the future. Furthermore, it allows the reconstruction of pedigrees for trees currently growing in the forest estate and demonstrating a range of responses to insect pests [11], pathogens [12–14] or other abiotic stresses [15, 16], providing valuable information for breeding for improved forest health. As the markers identified for pedigree reconstruction generate a unique profile for individual trees, these profiles can also, be used as a unique identifier to confirm clonal identity. Misidentification in seed orchards ranges from none detected [17] or low [18], to 15%–35% of ramets being mislabelled or misplaced [19]. The ability to accurately confirm the identity of individuals would allow for greater integrity of germplasm provenance for commercial producers of both seed orchard and clonal material. Traditionally, operational methods utilising markers, such as clone identification [20, 21] have made use of microsatellite markers (also called Simple Sequence Repeat (SSR) markers). While these methods can deliver a large amount of polymorphic information and discriminatory power with relatively few markers, the platforms for capturing the information are subject to loss of reliability over time due to differences in the materials and software used to capture the information both over time and between laboratories [10, 22].

An alternative to traditional microsatellite markers are the highly abundant, bi-allelic markers known as single nucleotide polymorphisms (SNPs) [23]. Estimated SNP abundance in pines varies in the literature and is dependent on the location of SNPs (genic versus non-genic regions) and the number of samples being genotyped. In *Pinus flexilis* E. James, transcriptomics-based resequencing reported 3.7 SNPs per kilobase [24]; in *P. taeda* L., Lu et al. [25] reported 11.5 SNPs per kilobase. Within *P. radiata*, SNP discovery via transcriptomic resequencing across 8 genotypes delivered 328,981 SNPs across 449,951 putative exons [26]. Even though more SNP markers are required than microsatellites for an equivalent level of discriminatory power, there is continual development of multiple platforms for capturing SNP genotypes, allowing for the cost-effective development of both high and low throughput assays [27]. In addition, the ability to generate a reproducible genotype from a given individual across time and between laboratories, is more robust [28].

The implementation of multiple molecular based technologies in forest tree breeding requires a sufficiently large genome-wide set of DNA markers. Such resources have only been realised through the advent of new DNA sequencing and genotyping technologies, which have allowed for cheaper and effective high throughput marker discovery. Development of a genome-wide SNP panel ideally involves whole genome resequencing of an appropriate reference population [29, 30], and mapping against a detailed reference genome [31, 32] to detect and order SNPs across the genome and assess their frequency within the population studied. However, whole genome sequencing and resequencing is often cost-prohibitive in large conifer megagenomes [33–36] The estimated genome size of *P. radiata* is in excess of 20 gigabases [37], distributed over 12 fairly evenly sized chromosomes [38].

Transcriptome-based SNP discovery has proven to be a successful alternative to whole genome resequencing. Such an approach assumes that gene-based SNPs will cover a sufficient proportion of the genome to enable the capturing of haplotype blocks that are needed for genomic predictions. It may even improve predictive ability either because the panel includes SNPs that cause trait variation, or a sufficient number of SNPs are in linkage disequilibrium with causative DNA sequence variants [39–43]. To date, a number of conifer programmes have developed transcriptome-based SNP resources for breeding applications, including Douglas-fir (*Pseudotsuga menziesi* Mirb.) [44], maritime pine (*Pinus pinaster* Aiton) [45] and radiata pine (*Pinus radiata* D.Don) [26]. There are a number of genotyping platforms suitable for assaying genome-wide markers including both fixed SNP arrays (Illumina [46], Affymetrix [47]) and genotype-by-sequencing (GBS) based approaches [48, 49]. We have chosen an exome capture GBS method that takes advantage of a previously developed transcriptome-based SNP resource in this species [26]. This method, which sequences portions of genes from across the genome was developed in *P. taeda* [50], and successfully developed for other large conifers, as well such as Norway spruce (*Picea abies*) [51] and Douglas-fir (*P. menziesii*) [52].

To date, no commercially available medium to high density SNP genotyping resources have been available for *P. radiata*. While some studies have published SNP resources for this species [53–55], these have usually been in the order of 10s or 100s of markers for association or diversity studies; none have satisfied the requirements for genomic selection in terms of sufficient numbers and distribution across the genome. This report describes the development of an exome capture probe panel for *P. radiata*, and initial evaluation of the efficacy of a subset of SNPs for pedigree reconstruction. This includes the development of a data processing pipeline that identified large numbers of high quality SNPs, suitable for application in several downstream genetic analyses. This SNP panel is now enabling the implementation of genomics in many facets of the New Zealand radiata pine breeding programme, to accelerate both advanced generation breeding and the development of improved clonal varieties.

## Materials and methods

### Plant material and DNA

We undertook a two-step design process to first test the efficacy of exome capture in *P. radiata*. In the first instance a small pilot set of samples (set 1) were selected to determine if the genotyping platform, Exome-capture GBS, could reproducibly identify biologically real, single-locus SNP variants, distinguishable from spurious sequencing errors. Experimentally, we made use of genotypes where variants had already been independently identified through RNA sequencing. Biologically, we also made use of parent/progeny trios to track Mendelian segregation and a maternally-derived haploid tissue, known as the megagametophyte, to confirm single-locus variants. The larger set 2, was designed to test and capture SNP variants across the New Zealand breeding populations of radiata pine and included linkage mapping populations, training populations, and putatively unrelated progenitor material.

#### Set 1 for pilot probe panel

A first set of 30 samples was used to obtain some preliminary performance metrics for the pilot probe panel, and consisted of: a) genotypes used to generate the exome templates [26] for capture probe design; b) ‘trios’, consisting of both parents and a single offspring to identify probes with non-Mendelian inheritance; c) six ‘duo’ parent and megagametophyte combinations to identify probes capturing paralogous regions; d) technical replicates of two unrelated genotypes to identify probes that did not yield repeatable results; and e) an additional replicate, prepared using an alternative DNA extraction protocol.

#### Set 2 for final probe panel

A second set of 1,345 samples was selected to evaluate more rigorously the final exome capture probe panel and the resulting SNP markers. This set included: a) two full-sib mapping populations, known as the quantitative trait loci (QTL) mapping population and the framework (FWK) mapping population [56]; b) a proof-of-concept genomic selection training population derived from two clonally replicated progeny trials, called POP2 and POP3 and described in Li et al. [55], consisting of 476 and 533 progeny respectively, to determine the efficacy of estimating genomic breeding values (GEBVs) [57]; c) 135 putatively unrelated trees, including the parents of POP2 and POP3, to estimate the minor allele frequencies (MAF) of SNP markers within the wider New Zealand *P. radiata* production population; and d) a single haploid megagametophytes from each of 104 of the 135 putatively unrelated trees.

### DNA extraction and quantification

Diploid needle tissue (100 mg) was homogenised using a Geno/Grinder^™^ 2000 (Spex SamplePrep, Metuchen, NJ, USA) and DNA extracted using the NucleoSpin^®^ Plant II DNA extraction kit (Machery-Nagel, Düren, GER), as described by Telfer et al. [58]. One of the replicates of parent 268345 was extracted using a modified CTAB method [58]. Haploid megagametophyte tissue was extracted using the same modified CTAB method. Seeds were soaked overnight on a moistened paper towel, and the megagametophytes dissected out the following day. Tissue was homogenized in 1 mL of CTAB buffer with two ceramic beads and sea sand, using a Bead Ruptor (Omni International, Kennesaw, GA, USA) at speed 5 for 20 seconds. DNA was then extracted as for needle tissue in Telfer et al. [58] and resuspended in 1x Tris HCl (pH 8.0) DNA quantifications were performed using a Qubit^®^ QuantIT dsDNA BR Assay kit and a Qubit^®^ Fluorometer (Thermofisher, Waltham, MA, USA). A minimum of 1 *μ*g of DNA was sent, at a minimum concentration of 20 ng/*μ*L, to the genotyping service provider RAPiD Genomics LLC (Gainesville, FL, USA).

### Development of genomic resources

#### Exome capture probe panel design

Exome capture probes for the pilot 80K panel were designed by RAPiD Genomics LLC, as per the steps described by Neves et al. [50]. RAPiD Genomics had found a marked increase in probe design efficiency when exon-intron boundaries had been accurately predicted. Therefore, a nucleotide BLASTn [59] was used to map a large set of *P. radiata* transcriptome contigs [26] onto the *P. taeda* genome v. 1.01e [35]. This resulted in 449,951 *P. radiata* exon templates and represented 46,342 *P. taeda* genomic scaffolds.

All possible probes (120 bp) were designed for the 449,951 predicted exon templates, yielding 1,164,086 potential probes. These were aligned to the *P. taeda* reference genome v.1.01e [35], and any probes mapping to multiple regions or chloroplast sequences were discarded. Probes aligning to regions containing either inserted or deleted DNA (INDELS) were also removed. The remaining 357,537 probes were aligned to the *P. radiata* transcriptome [26], and one probe per exon was selected. The resulting 37,118 probes covered regions containing 58,154 of the original 328,981 SNPs identified *in silico*, corresponding to 18,246 gene models. An additional 42,882 probes were included in the panel to bring the total number of probes up to 80,000. These additional probes consisted of 5,211 probes from *P. taeda* [50], and a further 37,671 probes, randomly selected from the remaining exons. The pilot 80,000 (80K) *P. radiata* exome capture probe panel designed by RAPiD Genomics LLC, therefore, consisted of 5,211 probes from *P. taeda*, and 74,789 probes designed from *P. radiata* transcriptomic sequence. Probe numbers from the pilot 80K panel were reduced to 48,914 (49K) after screening in the first 30 samples (set 1), with removal of monomorphic probes, probes that captured multiple loci, and those that were over-represented within the raw data. Inefficiencies in probe synthesis resulted in a total of 44,336 capture probes for use in the evaluation experiment (set 2 samples). Sequences for the two probe sets are included in the S1 and S2 Files.

### Genotyping

Genotyping was performed by RAPiD Genomics LLC using the exome capture genotype-by-sequencing (GBS) methods described by Neves et al [50, 60]. DNA was mechanically sheared, and libraries constructed from fragments in the range of 250-500 bp. These were hybridised to the capture probes, enriching the libraries for exons represented within the probe pool. The enriched libraries were sequenced from a single end using Illumina HiSeq 2000 sequencing (Illumina, San Diego, CA USA) to produce 100 bp reads. The raw reads were filtered by quality using FASTX-Toolkit (http://hannonlab.cshl.edu/fastx_toolkit), trimming bases with PHRED score below 20 from the 3’ end and removing reads shorter than 50 nt or containing more than 10% of bases below PHRED 20. The resulting reads were re-paired and mapped against a *P. taeda* reference v 1.01e [35] using Mosaik 2 Aligner [61] with non-default parameter -min 50 -mmp 0.05 -ls 500. For initial SNP discovery to produce a variant call file (.vcf), Freebayes [62] was utilized with default parameters.

### Filtering

Filters were applied to the raw genotype .vcf file, in the order described in Table 1 for the 80K probe panel, and Table 2 for the 49K probe panel.

**Table 1:** SNP filtering pipeline for 80K probe panel data.

Filter applied	Description
None	Raw calls post SNP discovery
Polymorphic haploids	Removed entire probes with heterozygous SNPs in more than 5% of the megagametophytes
Quality	A proprietary RAPiD Genomics quality score, removed SNPs Q < 10
Read depth per SNP	Removed SNPs with average read depth < 5X
Biallelic	Removed SNPs where more than two alleles were present
Allele ratio	Removed SNPs when the average ratio between the alternative and reference allele in the population was < 0.1 or > 0.9

**Table 2:** SNP filtering pipeline for 49K probe panel data.

Filter applied	Description
None	Raw calls post SNP discovery
Read depth per SNP	Removed SNPs if average depth across all individuals was < 10
Biallelic	Removed 2^nd^ (or 3^rd^) alleles if individual read depth was < 10, and reclassified SNP as biallelic. SNPs where the read depth for 2^nd^ (or 3^rd^) alleles was > 10 were classified as true triallelic and moved to a separate file.
Read depth per individual	Reclassified individual data points as missing if an individual had read depth < 10 for that SNP
Allele ratio	Removed individual data points where the ratio between reference and alternative alleles in a single individual genotype was < 0.1
Polymorphic haploids	Removed entire probes with heterozygous SNPs in >5% of megagametophytes
Monomorphic markers	Remove entire SNPs if monomorphic across our entire *P. radiata* sample set
MAF > 0.03	Remove entire SNPs if MAF > 0.03 in the 135 putatively unrelated individuals

### Analyses

#### Preliminary assessments in the 80K pilot study

The following quality metrics were used to assess the quality of SNPs generated in the 80K pilot study: call rate per sample, read depth, call rate per SNP, polymorphic SNPs per probe, minor allele frequency (MAF), heterozygosity within haploid (megagametophyte) samples, probes that successfully capture data and probe representation within the raw data. Mendelian inheritance in trios and duos, and reproducibility between replicate samples. Departure from Hardy-Weinberg expectation and average homozygosity were not considered suitable metrics for the sample size and composition of set 1.

#### SNP evaluation in the 49K panel study

The following quality metrics were used to assess the quality of SNPs generated in the 49K study: call rate per sample, read depth per sample, call rate per SNP, polymorphic SNPs per probe, minor allele frequency (MAF) in the unrelated 135 individuals, Mendelian segregation in the two mapping populations, linkage clustering in the one of the mapping populations, and reproducibility between replicate samples.

#### Probe distribution

To further examine the reliability of genotyping and the distribution of markers across the genome, we used JoinMap 4.1 [63] to check clustering of markers segregating in one full-sib linkage mapping population. Unreliable genotypes will be weakly linked or unlinked with other SNPs, with high quality data, the number of linkage clusters will be the same as the haploid chromosome (12) with few unlinked markers because of the high marker density of this panel. For a SNP to be assigned to a specific linkage group, it needed to be statistically associated with a least one other SNP with a log likelihood of odds ratio (LOD) of 9.0 or more.

#### Exclusion analysis for pedigree reconstruction

A subset of SNPs were selected from those identified using the 49K probe panel for testing the efficacy of pedigree reconstruction, with the view that this subset could potentially form the basis of a smaller targeted SNP panel. The criteria for selecting SNPs for pedigree reconstruction were a SNP call rate of greater than 0.75, and an observed minor allele frequency (MAF) between 0.35 and 0.5. In addition, selected SNPs did not deviate from Hardy-Weinberg equilibrium (HWE) as assessed using a Chi-square goodness-of-fit test [64]. SNP markers were only included if the p-value of this test was larger than 0.10. Finally, there was to be no evidence of linkage disequilibrium between selected SNP markers, with a required squared genotypic correlation (r^2^), used to measure composite linkage disequilibrium [65], of less than 0.02. We selected 704 SNPs from the pool of 80,160 informative SNPs that met the criteria mentioned above. The candidate parents were 117 putatively unrelated individuals with call rates > 0.6, and included the documented parents for both mapping populations. These mapping population parents were genotyped from multiple replicate samples and the individual sample with the highest call rate for each parent was used.

As previously described [10], an exclusion analysis approach was used to determine a trio relationship (a progeny and two candidate parents). For a given SNP, up to 4 possible genotype combinations could result during segregation from the two candidate parents. If the SNP genotype observed in an individual was not among the combinations possible from the parental genotypes, this SNP was considered an exclusion for that trio. SNP genotypes for a given progeny were compared against all the possible trio relationship combinations of the 117 putatively unrelated candidate parents. The number of exclusions were calculated for all 704 SNPs that satisfied the above-defined selection criteria. The trio relationship with the lowest number of exclusions was used to assign the most likely parents for an individual.

#### Pedigree reconstruction to confirm parentage in mapping populations

The pedigree of individuals in the two mapping populations had been previously confirmed [56]. To determine the minimum number of SNPs required to assign correct parentage in these populations, we randomly generated 60 sets of 110 SNPs from within the 704 SNP pool, to determine if 110 SNPs were sufficient to assign parentage correctly; exclusion analyses were performed to assess each of the sixty different panel combinations. Thereafter, we evaluated three of these panels to examine the potential impact of missing data on assignments, successively reducing the panels to contain 100, 90, 80, 70, 60 and 50 SNPs.

#### Clone identification

Clone identification analysis was used to differentiate a clonal relationship between two given offspring from the other potential relationships in this study, such as full-sibs (both parents in common), half-sibs (one parent in common) or unrelated. Individuals from the two mapping populations and the 117 unrelated individuals were used in this analysis. As multiple genotypes of the four mapping population parents (268345, 268405, 850055 and 850096) were available, the relationship between these multiple samples was defined as “ramet”. The relationship between the progeny within each mapping population was defined as “full-sibs”. The relationship between parents and progeny within each population was defined as “parent-progeny”. The relationship between the 135 unrelated individuals was defined as “unrelated”. For clone identification, an exclusion analysis was conducted to evaluate whether any two plants had identical genotypes across all the SNPs used. An exclusion meant that two individuals had different genotypes for at least one SNP. The number of exclusions were calculated for all possible combinations of candidate individuals within each defined relationship group across each of the 60 panels of 110 SNPs mentioned above. The profile of the number of exclusions were compared for each of the different relationships: ramet, full-sib, parent-offspring, and unrelated. SNPs where one or both genotypes were missing from a pair-wise comparison were excluded from the analysis. Analysis results were excluded if less than 80 of the 110 SNP comparisons were available.

## Results

### Evaluation of the 80K probe panel

**Fig 1:**
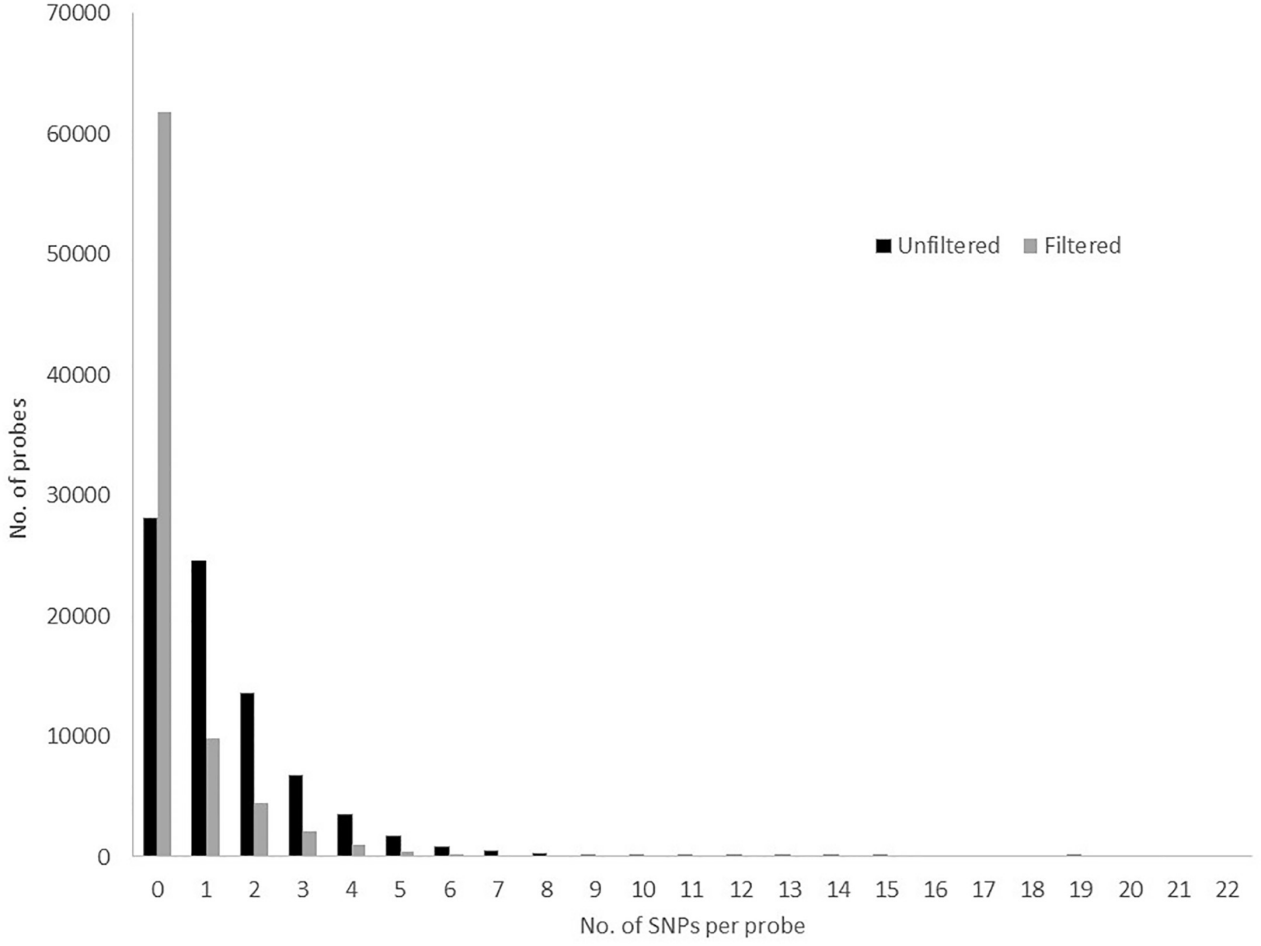
SNP markers per probe. Distribution of numbers of SNPs per probe for filtered and unfiltered data sets from the 80K probe panel.

The total number of probes from the pilot 80K panel that successfully captured genomic fragments from the *P. radiata* DNA samples in set 1 was 79,972 (99.97%). Initial SNP discovery within this small pilot study detected 284,058 SNPs (from 51,903 probes), which was reduced to 49,129 (from 18,222 probes) after filtering (Table 1) was applied. This represented capture of SNPs from 32,111 and 11,965 gene models from the unfiltered and filtered datasets, respectively. The average number of SNPs per probe was 1.34 and 0.43 for unfiltered and filtered datasets, respectively (Fig 1), ranging from 0 up to a maximum of 19. Of these SNP markers, only 149,632 (52.7%) of unfiltered SNPs and 18,702 (37.6%) of filtered SNPs achieved call rates exceeding 95% within the diploid samples. Reproducibility between replicate samples was 79.3–87.9% in the unfiltered dataset and 86.9–93.3% in the filtered dataset. This was also lower than seen in fixed array SNP assays such as the eucalypt 60K SNP chip [29], where reproducibility between replicate samples across the entire panel was 99.26–99.27% [10].

**Fig 2:**
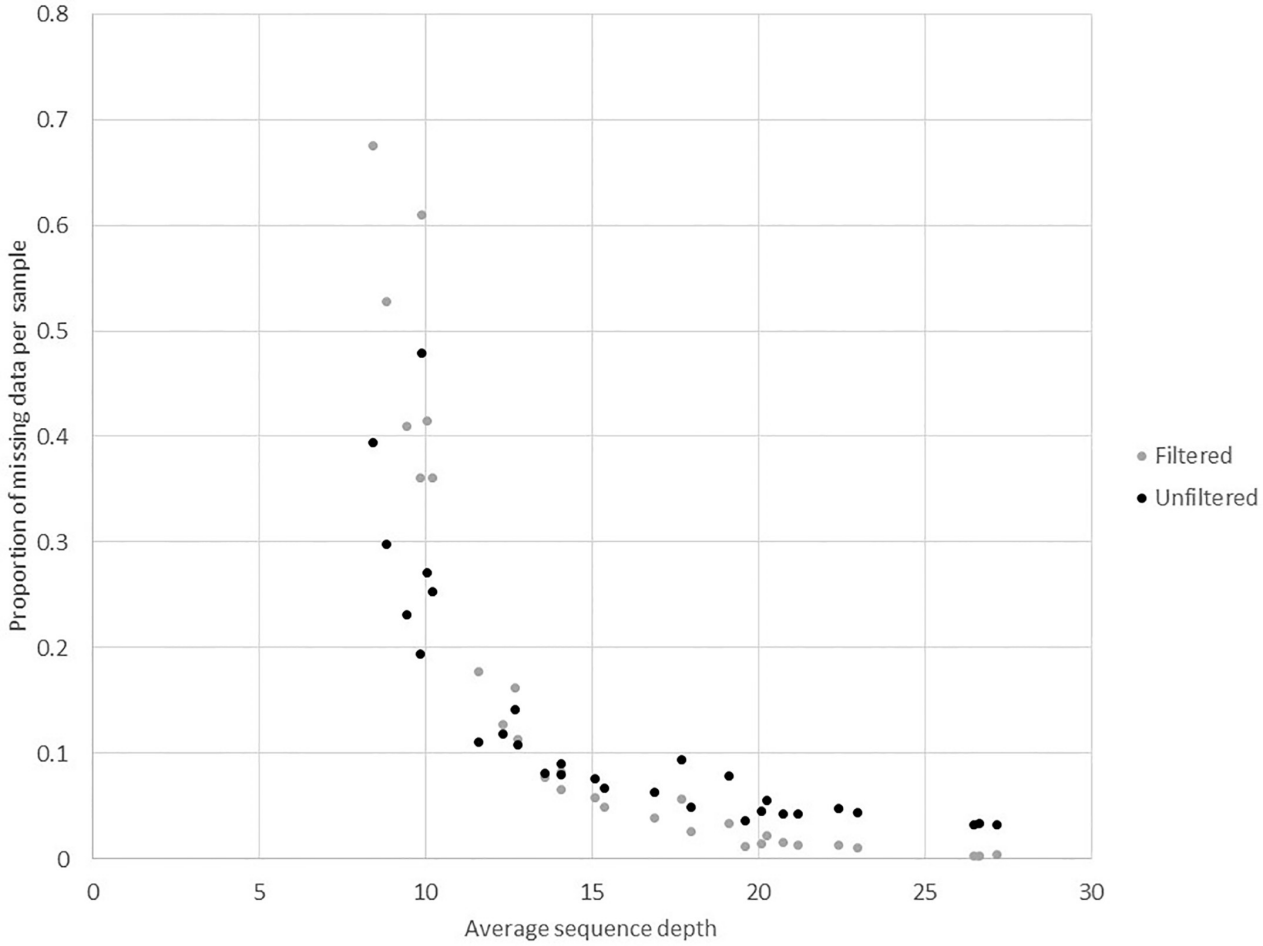
Impact of read depth. Relationship between average sequencing depth and the average proportion of missing data per sample for filtered and unfiltered data for the 80K probe panel.

We also examined the relationship between read depth and missing data points across the pilot 80K probe evaluation study (Fig 2), as read depth and missing data contributed to both reproducibility and pedigree reconstruction accuracy. These results indicated that for the unfiltered data, the proportion of missing data was largely invariant above 20X read depth—corresponding to approximately 4–5% missing data. When the read depth decreased to 15-20X, missing data increased to 10%, and further deteriorated to 20-50% when read depth dropped below 10X. For the filtered data, most samples with over 15X read depth had less than 5% missing data, while samples with a read depth below 15X had a wide range of missing data (8–68%). As a result, we adjusted the target read depth to 20X for subsequent genotyping using the 49K probe panel. Allele frequency distributions were estimated for the thirteen unrelated diploid samples in the filtered data set and showed a skewed distribution of MAFs toward low frequency. The estimated mean MAF was 0.16 ± 0.002, while the median MAF was 0.13.

Probe numbers from the initial 80K panel were reduced to 48,914 after testing in the first 30 samples, with removal of monomorphic probes, probes that captured multiple loci, and those that were overrepresented within the raw data. Inefficiencies in probe synthesis resulted in a total of 44,336 capture probes for use in the second evaluation experiment.

### Evaluation of the 49K probe panel

When evaluating the SNPs detected using the 49K probe panel, we observed that most samples had very little missing data. The median read depth across all loci was 66X, with a minimum of 6X and a maximum of 204X. Reproducibility across 6 replicates of tree 268345 was greatly improved in the refined 49K set due to further proprietary modifications to improve throughput and the capture of on-target sequence by Rapid Genomics LLC. In the filtered dataset, the pair-wise repeatability was 96% across the 775,307 SNPs captured in this tree’s genotype. A redesigned filtering pipeline, to first establish confidence in the data before using it to inform decisions to remove probes/SNPs, allowed more loci to remain within the SNP pool. Firstly, data quality metrics such as read depth and allele ratios were considered prior to the identification of triallelic or multi-locus markers. Secondly, the identification and removal of individual data points of low quality was preferred over complete removal of affected probes/SNPs from the entire dataset. A summary of the revised dataset is given in Table 3, with loss of only 5% of the genes and 6% of probes compared with 24% and 27%, respectively, under the original filtering pipeline.

**Table 3:** Application of the modified filtering to 49K probe dataset.

Filter applied	No. of genes	No. of probes	No. of SNPs
None	28,820	44,378	1,526,652
Read depth per SNP	28,813	44,367	1,525,238
Biallelic	28,813	44,363	1,410,361
Read depth per individual	28,813	44,363	1,410,361
Allele ratio	28,813	44,363	1,410,361
Monomorphic markers	28,813	41,822	849,519
Polymorphic haploids	27,318	41,822	781,434
MAF > 0.03 in 135 putatively unrelated individuals	27,318	41,822	80,160

### Population analyses

**Fig 3:**
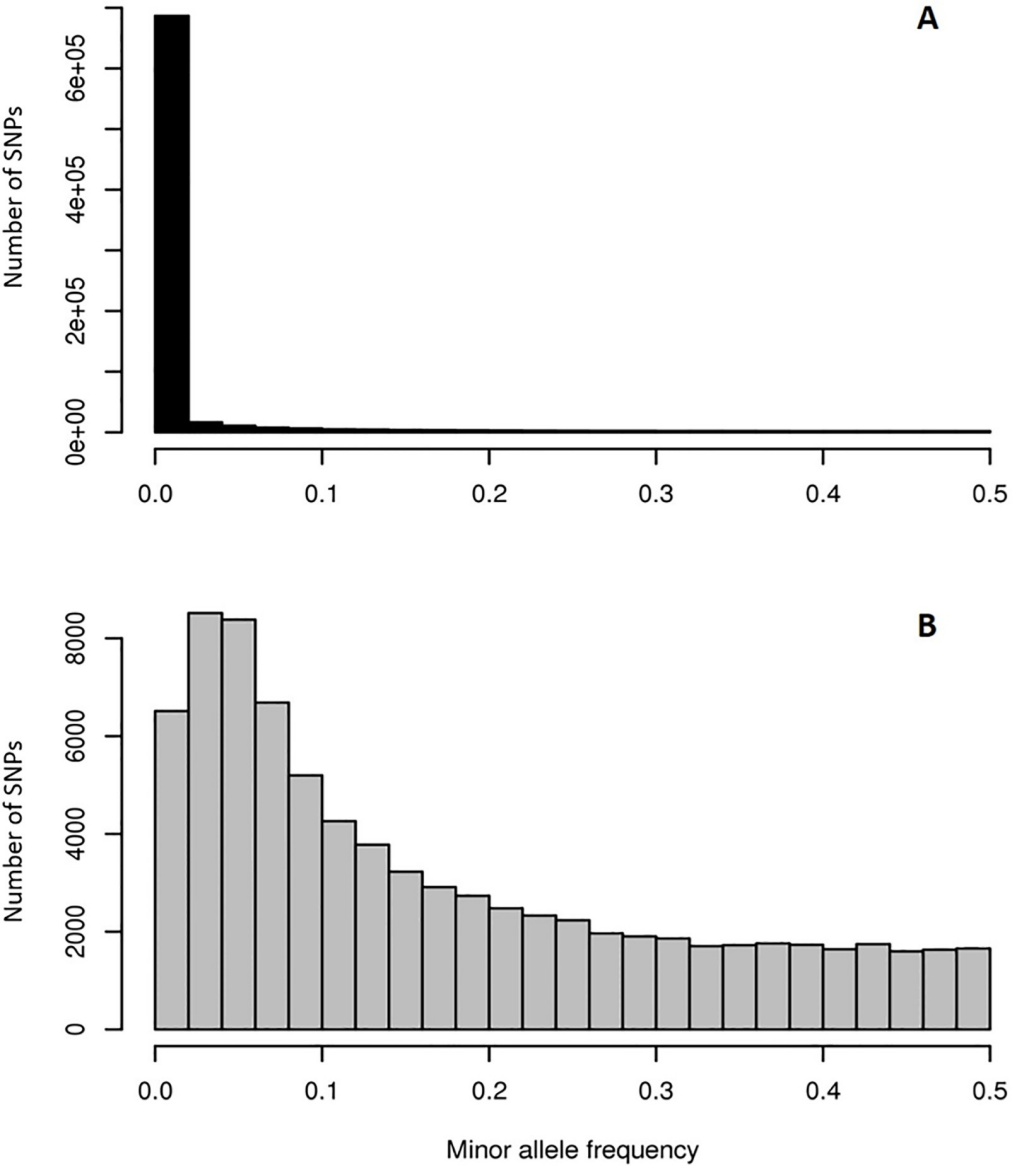
Minor allele frequency in 135 putatively unrelated individuals. Minor allele frequency distribution across A) the complete set of filtered SNPs generated from the 49K probe set and B) SNPs from the 49K probe set present at a MAF >0.03.

To deliver a dataset of SNP markers suitable for downstream analyses, we looked at the distribution of MAF within the filtered dataset for the 135 unrelated and parent individuals. The distribution of MAFs across the markers is still heavily biased to rare variants (Fig 3A), with 94% of the markers present as rare variants. Applying a more common MAF filter of greater than 0.05 would have left 70,872 SNPs in our dataset. However, we determined that to observe the minor allele in at least one individual in our training population (n = 1009) would correspond to a MAF of 0.03 in the unrelated and parent individuals (n = 135). Therefore, we applied a lower MAF filter of 0.03, resulting in a slightly larger set of 80,160 SNPs that could be used for downstream analyses (Fig 3B).

### Linkage analysis of probe distribution

After data quality filtering we used linkage analyses to check SNP data quality based on segregation and clustering in a full sib family consisting of n = 97 offspring. Linkage analysis was run on 21,386 markers, corresponding to 17,569 probes. Linkage analysis revealed twelve clusters of markers—equal to the haploid number of chromosomes in this species—with cluster sizes ranging from 1.356 − 2.200 x 103 SNPs per cluster. Only 204 SNPs (0.95%) could not be assigned to a cluster at LOD 9.0, however all SNPS clustered at 2.0 < LOD < 9.0.

### Pedigree reconstruction

**Fig 4:**
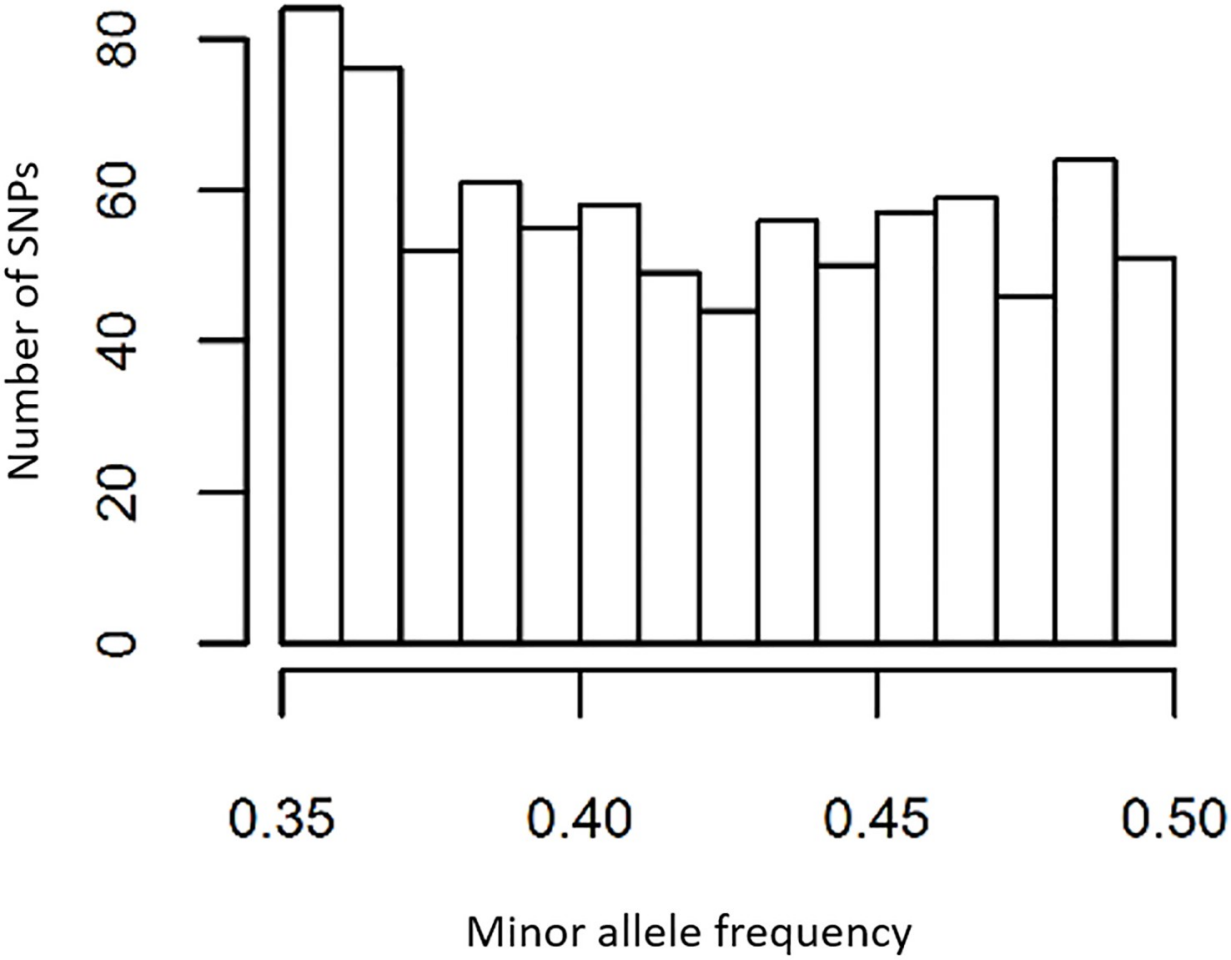
Minor allele frequency in 135 putatively unrelated individuals. Minor allele frequency distribution of 704 SNPs that passed the selection criteria for pedigree reconstruction.

Following the selection criteria for pedigree reconstruction, 704 SNPs were available for exclusion analysis, with MAF of the selected SNPs evenly distributed between 0.35 and 0.5 (Fig 4). We then explored the minimum number of SNPs required for a pedigree reconstruction panel. Tables 4 and 5 show the number of progeny assigned to candidate parents in the FWK and QTL mapping populations using the first 100, 90, 80, 70, 60 and 50 SNPs from 3 randomly selected panels of 110 SNPs that were randomly chosen from amongst the 494 SNPs. For the FWK mapping population, 70 SNPs or more were required to assign all 82 progeny to the true parents, with 1-5 false trio assignments across the three panels when SNP numbers decreased below 70. For the QTL mapping population, 80 SNPs or more were required to assign all 93 progeny to the true parents, with 1-7 false trio assignments when SNP numbers decreased below this threshold. However, further investigation showed that false assignments were associated with progeny where the call rate was much lower than the average for that given random sub-set of markers. Increased call rates with more robust genotyping platforms and additional markers to compensate for missing data will ensure sufficient SNP datapoints are available to make parental assignments. For most established platforms, error rates are well documented, allowing genotyping error rates to be factored into downstream analyses.

**Table 4:** Progeny assignment in FWK population.

Panel	Parent	No of SNPs in panel					
		100	90	80	70	60	50
Panel 1	True trio assignment	82	82	82	82	81	80
	False trio assignments	0	0	0	0	1	2
Panel 2	True trio assignment	82	82	82	82	81	77
	False trio assignments	0	0	0	0	1	5
Panel 3	True trio assignment	82	82	82	82	82	79
	False trio assignments	0	0	0	0	0	3

Number of progeny assigned to candidate parents in the FWK mapping population with true parents 850055 and 850096 using the first 100, 90, 80, 70, 60 or 50 SNPs from 3 panels of 100 SNPs that were randomly chosen from the 704 SNPs.

**Table 5:** Progeny assignment in FWK population.

Panel	Parent	No of SNPs in panel					
		100	90	80	70	60	50
Panel 1	True trio assignment	93	93	93	93	92	86
	False trio assignments	0	0	0	0	1	7
Panel 2	True trio assignment	93	93	93	93	93	89
	False trio assignments	0	0	0	0	0	4
Panel 3	True trio assignment	93	93	93	92	91	88
	False trio assignments	0	0	0	1	2	5

Number of progeny assigned to candidate parents in the QTL mapping population with true parents 268405 and 268345 using the first 100, 90, 80, 70, 60 or 50 SNPs from 3 panels of 100 SNPs that were randomly chosen from the 704 SNPs.

### Trio relationship analysis using 110 SNPs

To allow for some missing datapoints, we aimed to have a panel that contained 110 SNP markers, we generated 60 random SNP panels form the pool of 704 and assessed their discriminatory power. Almost all progeny in the QTL and FWK mapping populations were assigned to their true parents across all 60 panels (Table 6). In the QTL mapping population, one progeny was not assigned to its true parent in 5 out of 60 panels due to a low SNP call rate (0.66) in that individual. In the FWK mapping population, one individual was dropped in the analysis of five of the panels due to much lower call rates (0.45-0.49) for the SNPs represented in these panels.

**Table 6:** Parentage assignment accuracy.

Population	True Parent	No. of Progeny	Correct assignment(%)
QTL	268345	93	99.90
	268405	93	99.98
FWK	850055	82	99.90
	850096	82	99.90

Percentage of progeny assigned to true parents 850055 and 850096 in the FWK mapping population and true parents 268405 and 268345 in the QTL mapping population across 80 panels of 110 SNPs that were randomly chosen from the 494 SNPs that passed the TruSeq Amplicon assay design in Illumina.

### Clone identification

**Fig 5:**
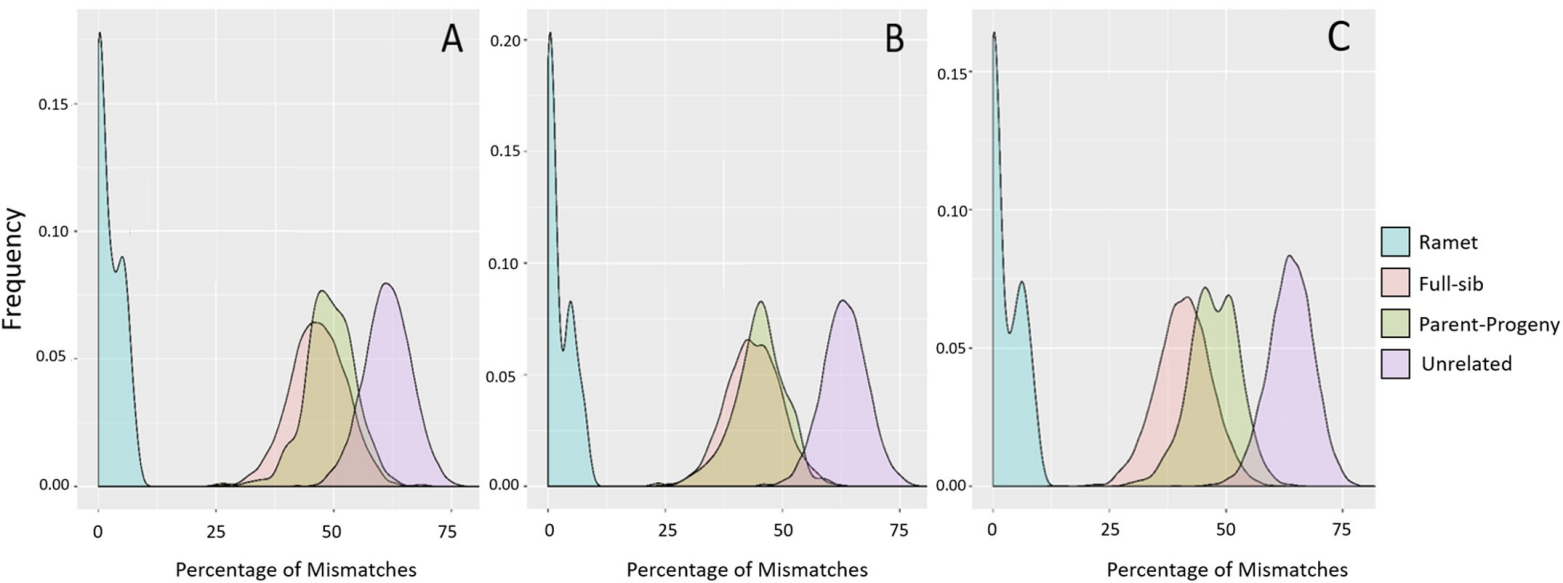
Profiles of mismatches in percentage for relationship groups. Ramet, full-sib, parent-offspring and unrelated in A) Panel 34 (maximum gap (16.17%) between ramet and the rest genotype groups); B) Panel 12 (medium gap (9.86%) between ramet and the rest genotype groups) and C) Panel 10 (minimum gap (3.59%) between ramet and the rest genotype groups).

**Fig 6:**
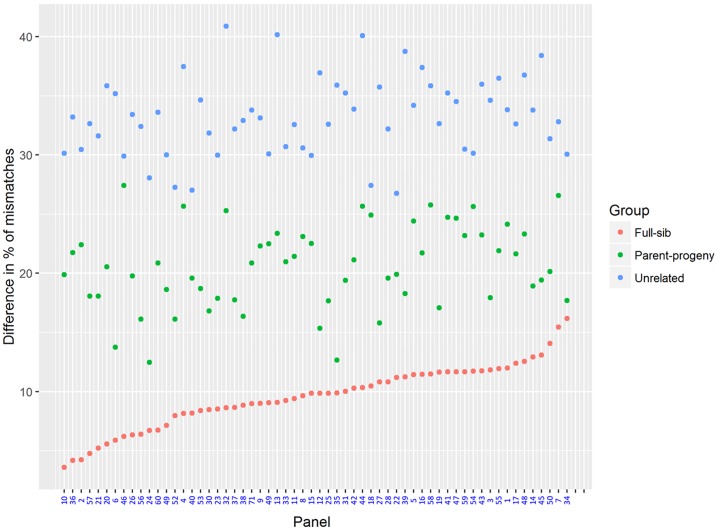
Variation in performance across 60 random 110-SNP panels. Gap between the maximum mismatches for ramets and the minimum mismatches for the full-sib relationship, the parent-offspring relationship and the unrelated relationship for the 60 random panels.

Fig 5 shows the profiles of percentage mismatches for the different relationship groups (ramet, full-sib, parent-offspring and unrelated) for the A) maximum, B) medium and C) minimum mismatch percentage between ramet and the rest of the genotype groups. Mismatched percentages ranged from 0-10.98% for the ramets and up to 36.05–82.96% for the putatively unrelated individuals (Table 7). As expected, after ramets, the full-sib relationship displayed the lowest minimum % mismatches. Therefore, to accurately differentiate ramets from full-sibs, an effective SNP pool should maximize the gap between the maximum % mismatches for ramets and the minimum % mismatches for full-sibs. Fig 6 shows panels ranked by the size of this gap. Panel 34 had the biggest gap between the mismatch profiles for ramets and full-sibs, which was 16.17% (Fig 6). This panel had a mismatch range of 0-7.87% for ramets, 24.04-68.42% for full-sibs, 25.56-68.52% for parent-offspring, and 37.93-77.67% for unrelateds. It is clear that overlap exists between the mismatch distribution of “putatively” unrelated individuals and the closer relationship classes, indicating a degree of relatedness exists in the progenitor material.

**Table 7:** The minimum and maximum of percentages of mismatches across 60 random panels for different relationships: Ramets, full-sibs, parent-offspring and unrelated.

Relationship	Minimum percentage of mismatches	Maximum percentage of mismatches
Ramet	0	5.00–10.98%
Full-sib	12.97-24.04%	59.04-69.05%
Parent-offspring	20.43-35.64%	56.60-71.70%
Unrelated individual	36.05-48.94%	74.29-82.95%

## Discussion

### Assay design and evaluation

The design process, which exploited the conservation in gene sequence between *P. radiata* and *P. taeda* to predict intron-exon boundaries, appears to have been highly successful, with over 99% of the original 80K probes successfully capturing genomic sequence from the prepared DNA samples. The original panel designed for *P. taeda* had demonstrated decreased capture efficiency associated with the presence of introns in probes [50]. The reference genome of *P. taeda* that was used to map the *P. radiata* transcriptome [26] was an incomplete assembly, still in 14.4 million scaffolds [35]. However, the annotation of that genome produced 50,172 gene models [66], indicating that our refined 49K probe set captures ∼54% of the predicted *P. taeda* genes. In addition, the linkage analysis performed in this study indicates that these gene-linked SNP markers are distributed approximately equally among the 12 linkage clusters, consistent with expectations for a species with 12 similarly sized chromosomes [38].

Within the numerous quality metrics we examined, the number of individual raw reads contributing to a variant being called had the greatest impact on data quality. Read depth impacted on the proportion of missing data which in turn influenced the reproducibility of genotypes between replicate samples and consistency of alleles within both parent/progeny trios and parent/megagametophyte duos. These results suggested that higher error rates were more likely in samples with low read depth due to unobserved heterozygotes being incorrectly assigned as homozygous genotypes. The decision to increase the target read depth thus allowed for improved retention of markers within the filtered pool. The impact on probe retention within the SNP pool was dramatic when the quality of individual SNPs was assessed first, and individual datapoints removed instead of entire SNPs or even whole probes. The most dramatic impact was the retention of ∼11,000 additional probes that had originally been removed due to the presence of heterozygous SNPs detected in the haploid samples. Closer examination revealed 281,391 (= 4,558 genes) of the putatively heterozygous SNPs had only a single read for the alternative allele. The presence of triallelic SNPs, a class we chose to exclude for further analysis, was also dramatically impacted by an initial filtering step based on read depth. Substitution error rates introduced by sequencing have been reported between 0.11% and 0.28% depending on the particular chemistry being used [67]. The implication is that as many as 3 bases per 1000 could be incorrectly assigned, therefore it is important to have sufficient number of observations to ensure variant calling is correctly applied. Confidence in the variant calling quality should be considered ahead of filtering steps that remove “biologically unsuitable” markers such as pseudo SNPs resulting from capture of paralogous loci.

Exome capture GBS has proven very efficient at capturing SNP markers, including low frequency ones. Over 94% of the markers captured in our large scale 49K evaluation panel were present at a MAF of less than 0.03. As new populations are genotyped, we will continue to capture additional variants and grow a SNP marker resource for downstream analyses. This is an advantage of sequencing-based approaches over fixed array SNP genotyping platforms which are limited to interrogating predefined SNP loci. Non-target or adjacent SNP markers and insertions/deletions, previously undocumented in a population, can also negatively impact the quality of genotypic scores generated from SNP arrays, which generally use shorter probes of 30-50 bases. The length of the exome capture probes (120 bp) gives improved specificity in terms of single-locus binding, as well as improved tolerance for undocumented polymorphism in the binding region. The impact of a rare SNP variant resource could be beneficial for a number of downstream applications and if exome capture GBS is used operationally, it is a resource that will continue to grow.

### Pedigree reconstruction

Under the SNP selection criteria developed for inclusion in pedigree reconstruction (MAF 0.35-0.5), only 704 (0.05%) were suitable for this purpose. It is possible that the low percentage of SNPs with intermediate frequency alleles, present in the representative 135 putatively unrelated individuals, could relate to the nature of exome capture as a source of SNP discovery, as a higher degree of conservation would be expected for genic regions. While a recent study in *P. taeda* showed that SNP markers are similarly prevalent in both genic (53%) and non-genic (42%) regions, the average allele frequency within the two classes was not discussed [25]. However, this study did report a median allele frequency of 0.14 after removal of SNPs below 0.05 in the population analysed. The attrition rate for SNPs that progress to useful SNP marker panels is high; in eucalypts, for example, ∼47 million SNP markers were screened before 60,904 were committed to a multi-species SNP array [29]. Over 500,000 SNPs were vetted to produce the OvineSNP50 bead chip [68]. Once the initial criteria for SNP selection had been established, the ability to select randomly from within the larger pool delivered an additional robustness in terms of satisfying the design requirements of potential downstream genotyping platforms. We observed no difference in discriminatory power between ramets and full-sibs between the 60 randomly selected 110 SNP marker panels (S1 Table), except in individuals with lower than average call rates. While it was possible to correctly assign parentage with as few as 70 markers in the case of the FWK population and 80 markers for the QTL population, the impact of potentially missing data in samples with low calls rate was mitigated by including an excess of markers within the final panel. The quality of the original tissue sample and method of DNA extraction can also affect the efficacy of genotyping [10]. Therefore, excess markers within the final panel confers additional robustness for commercial parentage or clone identification applications, where the source of the DNA sample is not always optimal.

### Clone identification

All 60 test panels performed well for pedigree reconstruction using exclusion analyses, although there was a marked difference in the discriminatory power between true ramets and full-sib relationships. However, the ability to distinguish material in the more distantly related classes was not as clear, indicating that a larger pool of SNPs, such as a medium density fixed array, may be required for pedigree reconstruction where hidden relatedness is present. All genotyping platforms, particularly those based on genotype-by-sequencing, can result in incorrect genotypes being called, particularly dependent on the variant calling and filtering algorithms employed to generate a variant call file [26, 69]. For fixed array SNP panels, the generally accepted error rate is that 99% of samples should provide data above the 0.1 GeneCall quality marker [70] for Illumina products. A test of the Affymetrix based arrays showed error rates ranging from 0.56–2.54% [71]. In MALDI-TOF based genotyping, the correlation between the alleles detected in a pooled sample compared to the individually genotyped samples was r^2^ = 0.886 [72]. To reduce the possibility of ramets and full-sibs being mistakenly assigned false relationships through genotyping errors, we looked for panels within the 60 randomly selected SNP sets that showed the greatest distance between the maximum number of mismatched alleles in the true ramets and the minimum number of mismatched alleles between full-sibs. In Fig 6, it is clear there was notable variation in this metric and that additional SNP markers will be required to fully elucidate complex pedigrees.

## Conclusion

We have successfully developed a high-density exome capture GBS probe panel for *P. radiata* from transcriptomic data, using a related reference genome. This resource enables interrogation of SNPs at a level that has not previously been reported for this species. The final 49K probe set captures an estimated 54% of the predicted gene models in *P. taeda* and was able to generate a large pool of SNPs (80,160) for downstream applications, such as genomic selection, pedigree reconstruction, clone identification, association studies and linkage mapping. The majority (94%) of the SNP markers identified are present at low to extremely low minor allele frequencies (MAF < 0.03). The potential of rare variants within populations for genomic predictions will need to be explored. The linkage clustering results indicate that most SNPs behave as discrete single locus markers consistent with Mendelian expectations. Precise genomic coverage is yet to be determined, but the initial results show good co-location and approximately similar distribution across all chromosomes.

Through the initial 80K pilot study and subsequent evaluation process we identified a minimum read depth of 20X to accurately distinguish sequencing errors from genuine SNP markers. Improvements made to the capture protocols are now achieving an average read depth of 60X with the same initial sequencing strategy. We also developed filtering pipelines which focused on data quality prior to removing probes or individual SNPs which showed evidence of capturing multiple loci or had more than two alleles present in a population of interest. This has increased the number of gene models included within the filtered dataset from 22,740 to 27,260. The result is that a greater portion of the genome is being captured and is available for use in downstream analyses.

We successfully identified 704 SNP markers from within the pool of SNPs which met our criteria for pedigree reconstruction and clone identification. We were able to assign parentage correctly in two large full-sib mapping populations, with as few as 70 SNP markers in most instances. The impact of missing datapoints in progeny or parents with low call rates was most likely the cause of the observed parental misassignments. Therefore, we recommend a set of 110 SNPs that can be used by our industry stakeholders for pedigree reconstruction and clone identification for radiata pine in New Zealand, to be used for the development of an operational SNP-based parentage assay using an affordable, scalable, and robust genotyping platform. Our results indicate that this set of SNPs will be robustly effective for distinguishing clonal from first-degree (i.e. parent-offspring and full sib) relationships. However, more complex pedigree reconstruction tasks (e.g. classification into different relationship categories and/or discrimination of more distant relatives from nominally unrelated individuals) will likely require using much larger numbers of SNPs and more sophisticated methodology to account for genotyping error and rates of false positive and false negative classifications. This will most likely be achieved through design of a medium density fixed-array genotyping resource using these results. Full pedigree information will become achievable for open-pollinated and mixed-seedlot trees, provided all possible candidate parental genotypes are available for comparison with potential progeny genotypes. Generating a complete (or near-complete) database of potential parents reflective of our breeding and production populations will be a priority going forward. In addition to pedigree reconstruction, the clonal *P. radiata* training populations, POP2 and POP3 were genotyped with the optimised 49K probe panel, and first proof of concept genomic estimated breeding values are presented in [57].

The benefit of developing an exome capture GBS panel for *P. radiata* is the wealth of additional information that is captured. Rare variants, seldom captured in fixed arrays, are available for analysis. Flanking genomic sequence is also available, which can prove beneficial in the development of low density SNP panels that are more suitable for pedigree reconstruction and routine clone identification. Well-evaluated SNPs can similarly be converted to robust fixed array medium density SNP panels, suitable for cost-effective screening of 10,000s of seedlings. These panels will enable New Zealand’s radiata pine breeding programmes to move towards the implementation of genomic selection, increasing the delivery of genetic gain through shortened generation intervals, which is increasingly important to select for resilience to new abiotic and biotic threats.

## Supporting information

S1 Table No of progeny that were assigned to the true parents in the QTL and FWK mapping populations for 60 SNP panels. The total number of progeny that were used in the analysis was 93 in the QTL mapping population and 82 in the FWK mapping population.
(DOCX)

S1 File 80k probe set. The sequence of probes designed for 80K pilot.
(FA) Click here for additional data file.

S2 File 49k probe set. The sequence of probes designed for 49K pilot.
(TXT)
