# Supplementary material for: A high-density exome capture genotype-by-sequencing panel for forestry breeding in Pinus radiata
Source: PLoS One. 2019 Sep 30;14(9):e0222640. doi: 10.1371/journal.pone.0222640 (PMC6768539; doi:10.1371/journal.pone.0222640)
Supplement: S1 Table — The total number of progeny that were used in the analysis was 93 in the QTL mapping population and 82 in the FWK mapping population. (DOCX) [file pone.0222640.s001.docx]

****SUPPLEMENTAL DATA****

**Supplemental table 1. No of progeny that were assigned to the true parents in the QTL and FWK mapping populations for 60 SNP panels. The total number of progeny that were used in the analysis was 93 in the QTL mapping population and 82 in the FWK mapping population**

| Panel | QTL mapping population | |  | FWK mapping population | |
| --- | --- | --- | --- | --- | --- |
|  | 268345 | 268405 |  | 850055 | 850096 |
| 1 | 93 | 93 |  | 82 | 82 |
| 2 | 93 | 93 |  | 82 | 82 |
| 3 | 93 | 93 |  | 82 | 82 |
| 4 | 93 | 93 |  | 82 | 82 |
| 5 | 93 | 93 |  | 82 | 82 |
| 6 | 93 | 93 |  | 82 | 82 |
| 7 | 92^§^ | 93 |  | 82 | 82 |
| 8 | 93 | 93 |  | 82 | 82 |
| 9 | 93 | 93 |  | 81^ξ^ | 81^ξ^ |
| 10 | 93 | 93 |  | 82 | 82 |
| 11 | 93 | 93 |  | 82 | 82 |
| 12 | 93 | 93 |  | 82 | 82 |
| 13 | 93 | 93 |  | 82 | 82 |
| 14 | 93 | 93 |  | 82 | 82 |
| 15 | 93 | 93 |  | 82 | 82 |
| 16 | 93 | 93 |  | 82 | 82 |
| 17 | 93 | 93 |  | 81^ξ^ | 81^ξ^ |
| 18 | 93 | 92^§^ |  | 82 | 82 |
| 19 | 93 | 93 |  | 82 | 82 |
| 20 | 93 | 93 |  | 82 | 82 |
| 41 | 93 | 93 |  | 82 | 82 |
| 42 | 93 | 93 |  | 82 | 82 |
| 43 | 93 | 93 |  | 82 | 82 |
| 44 | 93 | 93 |  | 82 | 82 |
| 45 | 93 | 93 |  | 82 | 82 |
| 46 | 93 | 93 |  | 82 | 82 |
| 47 | 93 | 93 |  | 82 | 82 |
| 48 | 92^§^ | 93 |  | 82 | 82 |
| 49 | 92^§^ | 93 |  | 82 | 82 |
| 50 | 93 | 93 |  | 82 | 82 |
| 51 | 93 | 93 |  | 82 | 82 |
| 52 | 92^§^ | 93 |  | 82 | 82 |
| 53 | 93 | 93 |  | 82 | 82 |
| 54 | 93 | 93 |  | 82 | 82 |
| 55 | 93 | 93 |  | 82 | 82 |
| 56 | 93 | 93 |  | 82 | 82 |
| 57 | 93 | 93 |  | 82 | 82 |
| 58 | 93 | 93 |  | 82 | 82 |
| 59 | 93 | 93 |  | 81^ξ^ | 81^ξ^ |
| 60 | 93 | 93 |  | 82 | 82 |
| 61 | 92^§^ | 93 |  | 82 | 82 |
| 62 | 93 | 93 |  | 82 | 82 |
| 63 | 93 | 93 |  | 82 | 82 |
| 64 | 93 | 93 |  | 82 | 82 |
| 65 | 93 | 93 |  | 81^ξ^ | 81^ξ^ |
| 66 | 93 | 93 |  | 82 | 82 |
| 67 | 93 | 93 |  | 82 | 82 |
| 68 | 93 | 93 |  | 82 | 82 |
| 69 | 93 | 93 |  | 82 | 82 |
| 70 | 93 | 93 |  | 82 | 82 |
| 71 | 93 | 93 |  | 82 | 82 |
| 72 | 93 | 93 |  | 82 | 82 |
| 73 | 93 | 93 |  | 82 | 82 |
| 74 | 93 | 93 |  | 82 | 82 |
| 75 | 93 | 93 |  | 82 | 82 |
| 76 | 93 | 93 |  | 82 | 82 |
| 77 | 93 | 93 |  | 81^ξ^ | 81^ξ^ |
| 78 | 93 | 93 |  | 82 | 82 |
| 79 | 93 | 93 |  | 82 | 82 |
| 80 | 93 | 93 |  | 82 | 82 |

^§^ Parent of one progeny (QTL_90_6) in the QTL mapping population was not assigned to 268345 due to low call rate. The call rate of 494 SNPs for progeny QTL_90_6 was 66%.

^ξ^ Progeny FWK_3_53 not included in the analysis in the panel due to low call rate (below 60%) for those particular randomly selected sets of SNPs.
